# Thermo-Kinetic Framework for TGA Curve Modeling and Evaporation Enthalpy Determination in Composite Materials: The Case of Bone-Derived Hydroxyapatite

**DOI:** 10.1021/acs.jpcb.6c01424

**Published:** 2026-06-24

**Authors:** Leon R. Bernal-Alvarez, Ivan Santamaria-Holek, Jose Luis Rivera-Armenta, Cristian F. Ramirez-Gutierrez

**Affiliations:** † Universidad Nacional Autónoma de México, 7180 Centro de Física Aplicada y Tecnología Avanzada, Campus Juriquilla, Querétaro C.P. 76230, México; ‡ UMDI-Facultad de Ciencias, Universidad Nacional Autónoma de México, Campus Juriquilla, Juriquilla, Querétaro 76230, Mexico; § 149207 Tecnológico Nacional de México/Instituto Tecnológico de Ciudad Madero, Prol Bahía de Aldahir y Av. de las Bahías S/N Parque de la pequeña y Mediana Industria, Altamira, Tamaulipas 89600, Mexico; ∥ Department of Ecology, Environment and Geoscience, Umea Universitet, Umea 90187, Sweden

## Abstract

A thermo-kinetic model based on nonequilibrium thermodynamics is presented to describe the sequential thermal degradation of multicomponent composite materials under nonisothermal conditions. The formulation is derived from the Gibbs free energy balance and the entropy-production principles, leading to a system of coupled differential equations in which mass-loss kinetics are governed by their conjugate thermodynamic forces and the externally imposed heating rate. Within this framework, the linear relations between fluxes and forces are characterized by generalized phenomenological coefficients defined in the Gibbs free energy representation, which are appropriate for experimentally controlled intensive variables. The model was applied to a biogenic hydroxyapatite composite obtained from bovine bone powder and validated by thermogravimetric analysis (TGA) at heating rates of 3, 5, 7, 25, 50, 75, and 100 °C/min. The model successfully reproduces the characteristic thermal degradation stages of the composite in inert conditions, enabling the determination of the apparent evaporation enthalpies of each constituent and revealing their dependence on the applied heating rate. Analysis of these trends identifies a quasi-static regime at low heating rates. For the specific case of the biohydroxyapatite system studied here, a heating rate of 5 °C/min lies within the quasi-stationary low-ramp regime and is therefore suitable for obtaining reliable apparent enthalpies. Owing to its general formulation, the model is applicable to composite systems with an arbitrary number of components, providing a physically grounded framework that extends the interpretation of TGA measurements beyond empirical curve fitting toward a consistent thermodynamic and physicochemical description of thermal degradation processes.

## Graphical abstract

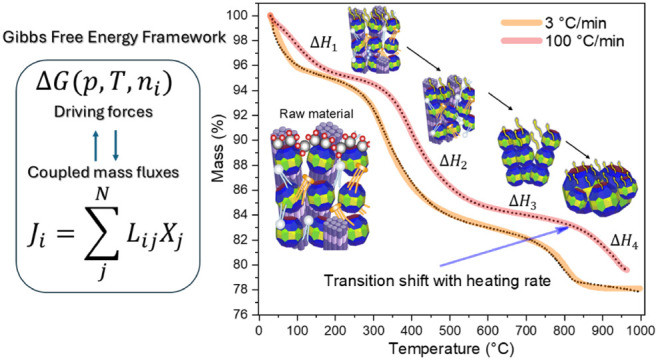

## Introduction

Thermogravimetric analysis (TGA) is a well-established technique for evaluating the thermal stability, composition, and degradation behavior of composite and complex materials.^1–3^ By monitoring mass changes as a function of temperature under controlled atmospheres, TGA enables the identification of key thermal events, including dehydration, decomposition of organic constituents, and mineral transformations.^4–7^ From a thermodynamic perspective, these mass variations reflect the macroscopic response of coupled heat and mass transfer processes evolving under nonequilibrium conditions.

TGA has been extensively applied to both synthetic and naturally derived composites^8,9^ including hydroxyapatite-based biocomposites^10,11^ marine shells, and lignocellulosic materials.^12–15^ In such heterogeneous systems, the technique is particularly valuable due to its sensitivity to differences in the thermal stability of constituent phases. In bioinspired and biocomposite materials, TGA also enables the assessment of properties such as moisture content, organic fraction, and structural organization, where thermal degradation patterns serve as indirect indicators of crystallinity and structural integrity.^16^ Beyond compositional characterization, TGA has been increasingly employed to estimate kinetic parameters and enthalpies associated with mass-loss processes, providing indirect access to thermophysical properties that govern phase interactions and influence material behavior during synthesis and thermal processing.^17–20^

Despite its versatility, the quantitative interpretation of thermogravimetric (TG) data remains challenging. Experimental measurements are inherently sensitive to instrumental configuration, heat and mass transfer limitations, and gas flow conditions,^21–23^ leading to significant variability across reported results. This variability is not merely experimental scatter but reflects the intrinsic nonequilibrium nature of thermally activated processes. An inappropriate selection of heating rate may also result in overlapping of distinct degradation processes, complicating their interpretation. In addition, TGA provides access to the reaction conversion associated with individual processes, which can be used for kinetic analysis.^24^ In practice, kinetic analyses are often limited to identifying characteristic temperatures using derivative-based methods applied to TG curves. Despite their practical usefulness in extracting effective kinetic parameters and correlating peak temperatures with activation energies and empirical approaches remain fundamentally limited.^25^ More advanced approaches typically rely on semiempirical models that reproduce the overall mass-loss profile but lack a mechanistic basis and fail to resolve interactions between overlapping degradation pathways.^26,27^ Consequently, these methods may obscure the underlying physicochemical processes and restrict predictive capability, particularly in multicomponent systems.

These limitations are especially evident in multicomponent systems such as bone, where organic and inorganic phases undergo complex, partially overlapping transformations,^28–30^ Variations in composition, microstructure, and thermal history lead to markedly different degradation trajectories, often resulting in inconsistent interpretations of thermal stability.^31^ As a result, TG curves are frequently treated as qualitative fingerprints rather than as a basis for predictive modeling.^32,33^ This lack of a rigorous framework undermines reproducibility and complicates the extraction of physically meaningful thermodynamic and kinetic parameters.

To overcome these limitations, this work introduces a thermokinetic framework based on irreversible thermodynamics for the analysis of composite degradation kinetics. The proposed model is designed to resolve the sequential thermal decomposition of multicomponent systems, with particular emphasis on the TG response of bone powder. In this system, distinct mass-loss events associated with moisture evaporation and the degradation of organic constituents (e.g., lipids and proteins) are explicitly described as coupled processes evolving with temperature. Unlike conventional approaches based on empirical curve fitting, the model incorporates cross-coupling interactions between components, enabling a mechanistically consistent interpretation of the observed mass-loss behavior.

The formulation is developed in the Gibbs free-energy representation, which is naturally suited to the constant-pressure conditions of TG experiments and provides a direct and physically consistent description of the system evolution. Within this framework, thermodynamic forces and fluxes are defined in terms of Gibbs free-energy variations, rather than through the classical entropy-based representation. As a result, the associated flux–force pairs and their proportionality coefficients differ from the standard Onsager formulation^34,35^ although the overall structure of the theory remains consistent with the Onsager-Prigogine formalism.^36^ This approach is supported by previous studies demonstrating that irreversible thermodynamics provides a rigorous basis for deriving evolution equations of mesoscopic systems from entropy-production and free-energy balances under nonequilibrium conditions.^37^

The governing equations are derived from entropy-production, with mass fluxes expressed as linear functions of their conjugate thermodynamic forces. The interactions between degradation pathways are captured through phenomenological transport coefficients that account for both direct and cross-coupled contributions among the constituent components. These coefficients enable the incorporation of kinetic interdependencies and interaction effects, including the formation of intermediate and final phases. Consequently, the proposed framework provides a physically grounded basis for describing degradation pathways and for improving the accuracy of thermodynamic quantities such as evaporation enthalpies.

## Materials and Methods

### Sample Preparation

The preparation of the raw biogenic hydroxyapatite (BHA) powder was executed following the procedure outlined by Bernal-Alvarez et al.^38^ According to this methodology, bovine cortical bone was initially sectioned into 5 cm-thick slices using a meat saw. A primary cleaning step was performed to obtain a relatively clean powder surface. This involved repeatedly boiling the slices in distilled water for 1 h, followed by drying in an Ecocell oven at 90 °C for 1 h. Subsequently, the samples underwent a secondary hydrothermal treatment in a Terlab autoclave using distilled water at 120 °C and 103 kPa. The final step involved mechanically reducing the size of the treated bone disks by crushing and grinding, yielding a fine powder with a particle-size distribution constrained to be less than 74 μm.

### Thermogravimetric Characterization

TG measurements were carried out using a TA Instruments Q600 simultaneous thermal analyzer (SDT, New Castle, DE, USA), operated in a side-loading horizontal configuration (Figure 1). The instrument is equipped with platinum/platinum–rhodium thermocouples and features a balance sensitivity of 0.1 μg.

**Figure 1:**
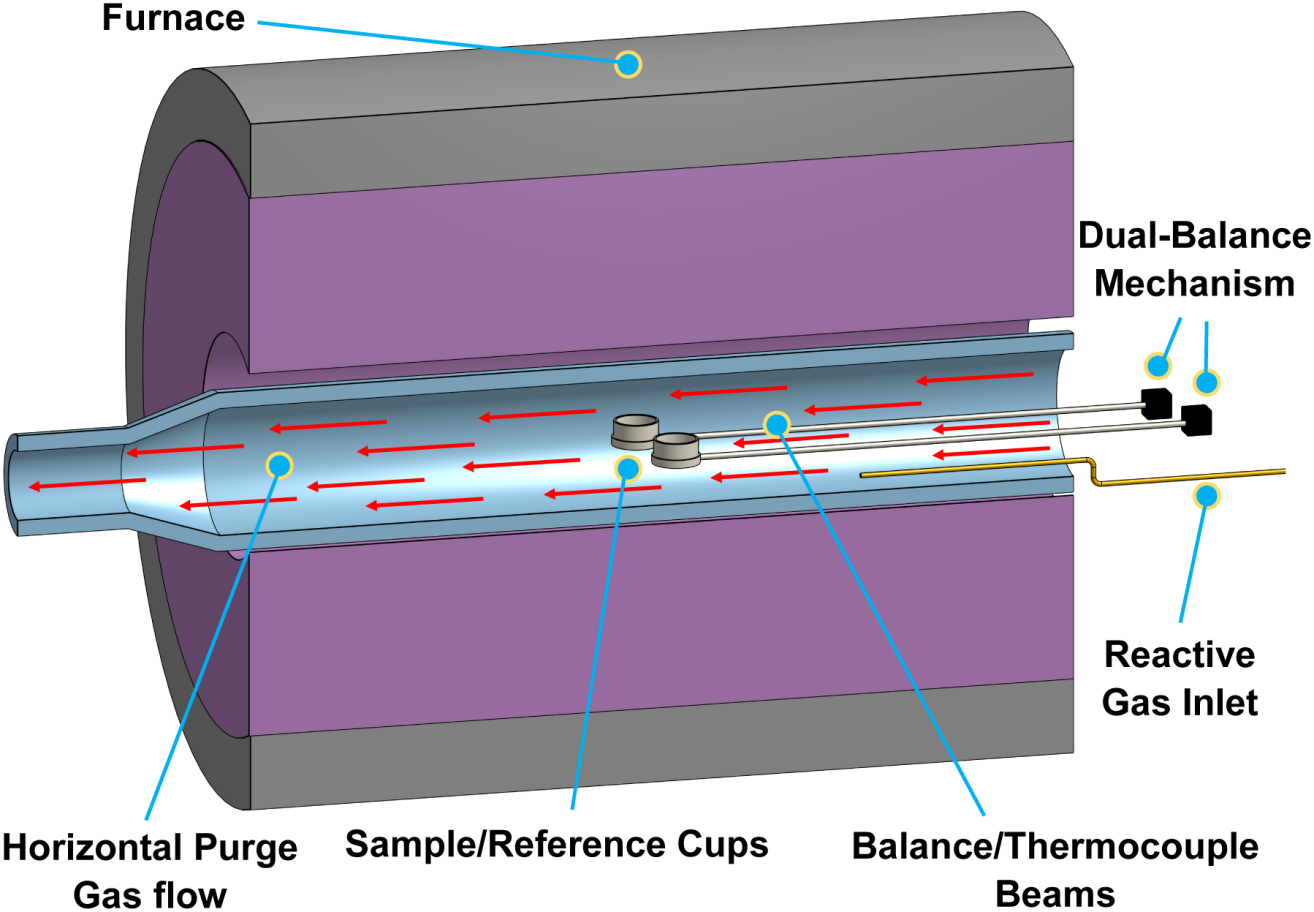
Side loading configuration scheme of TG equipment.

This geometry, in which the furnace is oriented horizontally and encloses the sample and reference holders positioned on the balance/thermocouple beams, was selected to enhance signal quality for kinetic measurements. In this configuration, a controlled purge gas flows horizontally across the crucibles, promoting laminar, nonturbulent conditions at the sample surface. Such flow characteristics are critical to minimizing noise in the mass-change signal and preserving well-defined concentration gradients, thereby improving the reliability of kinetic parameter estimation.

Nonisothermal experiments were conducted to investigate the thermal degradation kinetics of bone powder and to support the development of the proposed kinetic model. All measurements were performed under an inert nitrogen (N_2_) atmosphere with a constant flow rate of 100 mL/min. Samples of 12 ± 2 mg were placed in alumina crucibles and heated from room temperature to 1000 ^◦^C at heating rates of 3, 5, 7, 25, 50, 75, and 100 ^◦^C/min.

### Morphological Images by SEM and HR-SEM

*Ex-situ* images were acquired both before and after thermal treatment (referred to as bone powder and BHA, respectively). The bone powder was analyzed using a JEOL JSM-7401F scanning electron microscope (SEM) operating at an acceleration voltage of 20 kV, whereas the BHA samples were examined with a Hitachi SU8230 high-resolution scanning electron microscope (HR-SEM) at 1.0 kV. Both backscattered electron (BSE) and secondary electron (SE) signals were used for imaging.

## Theoretical Framework

### Thermogravimetric Analysis

From a theoretical standpoint, the thermogravimetric signal represents the temporal evolution of the mass function *m*(*T,t*) under nonequilibrium conditions imposed by a controlled thermal program and a defined gaseous atmosphere. Under dynamic heating, the transformations recorded by the TG signal do not correspond to equilibrium phase transitions, but rather to kinetically controlled processes governed by the interplay between reaction kinetics, heat transfer, and mass transport of volatile species. In this regime, the rate of mass loss is directly related to the temperature-dependent reaction rate constant *k*(*T*), which is commonly described by an Arrhenius-type expression,^39–41^ Consequently, the position, shape, and intensity of degradation steps in TG curves are intrinsically dependent on experimental parameters such as heating rate, sample mass, and purge gas conditions. This dependence confers upon TGA an essentially comparative and methodological character rather than an absolute thermodynamic one.^42^

Instrumental configuration and gaseous environment define the effective thermodynamic and kinetic boundary conditions of a thermogravimetric experiment. Current TG implementations, such as top-loading, bottom-loading, or side-loading geometries (e.g., Figure 1), as well as single and dual-pan designs, differ in balance positioning, heat-transfer efficiency, and gas-flow distribution around the sample.^5,22,43–45^ These differences may introduce systematic variations in buoyancy correction, baseline stability, thermal gradients, and hydrodynamic behavior, leading to measurable shifts in apparent onset temperatures, second-derivative (DDTG) peak positions, and mass-loss rates. In parallel, the selection of the purge atmosphere plays a decisive role, as the use of an inert gas suppresses oxidative side reactions and enables the continuous removal of volatile decomposition products.^7^ This sustained elimination prevents the establishment of chemical equilibrium in the vicinity of the sample and enforces an irreversible degradation pathway consistent with the Le Châtelier-Braun principle.^46^ Under these conditions, the Gibbs free-energy change associated with degradation remains negative throughout the process, preserving the directionality of mass loss and enhancing the reproducibility of characteristic thermal events. Consequently, both instrumental configuration and atmospheric control must be explicitly considered when interpreting TG data or comparing results across different experimental setups. Within this framework, the TG signal can be consistently interpreted as a macroscopic manifestation of underlying irreversible processes, thereby providing the experimentally constrained basis required for the formulation of the nonequilibrium thermodynamic model developed in the following section.

### Modeling the Thermal Degradation Kinetics

#### Gibbs Free Energy and Entropy Production

The total entropy change of the system can be decomposed as

1 $$dS=d_{e}S+d_{i}S$$

where *d*_*e*_ *S* is the entropy exchanged with the surroundings and *d*_*i*_ *S* ≥ 0 is the entropy produced internally by irreversible processes.

Since the TG experiment is carried out at constant pressure, the Gibbs free energy representation is the natural thermodynamic description. For the degrading sample (superscript (*s*)), one has,

2 $$dG^{\left(s\right)}\left(T,n_{i}\right)=-S^{\left(s\right)}dT+\overset{N}{\underset{i=1}{\sum }}\mu_{i}^{\left(s\right)}dn_{i}$$

The external atmosphere and furnace act as a heat bath or reservoir. In the present experiment, however, this reservoir is not isothermal in the static sense, since its temperature is externally imposed through the heating program,

3 $$T=T\left(t\right),,v≡\frac{dT}{dt}$$

The reservoir is assumed to be sufficiently large so that its intensive reference properties are not modified by the degradation of the sample. Accordingly, during each infinitesimal step, the reservoir contribution or heating bath (superscript (*HB*)) can be written as

4 $$dG^{\left(HB\right)}=-S^{\left(HB\right)}dT+\overset{N}{\underset{i=1}{\sum }}\mu_{i}^{\left(HB\right)}dn_{i}$$

The entropy-production is associated with the thermodynamic mismatch between the sample and the reservoir. Therefore,

5 $$Td_{i}S=dG^{\left(HB\right)}-dG^{\left(s\right)}\geq 0$$

Substituting eq 2 and 4 into eq 5, one obtains

6 $$T{\;d}_{i}S=\left(S^{\left(s\right)}-S^{\left(HB\right)}\right)dT-\overset{N}{\underset{i=1}{\sum }}\left(\mu_{i}^{\left(s\right)}-\mu_{i}^{\left(HB\right)}\right)dn_{i}$$

This expression shows that the entropy-production contains two contributions: a thermal contribution associated with the imposed temperature ramp, and a chemical contribution associated with the exchange or loss of material. Defining

7 $$\Delta S≡S^{\left(s\right)}-S^{\left(HB\right)}$$

and the generalized chemical affinities

8 $$A_{i}≡\mu_{i}^{\left(s\right)}-\mu_{i}^{\left(HB\right)}$$

(Equation 6) becomes

9 $$T{\;d}_{i}S=\Delta S\;dT-\overset{N}{\underset{i=1}{\sum }}A_{i}\;dn_{i}$$

To express the result in terms of the experimentally measured masses, we use *m*_*i*_ = *W*_*i*_ *n*_*i*_, where *W*_*i*_ is the molar mass of the *i*th component. Introducing the affinity per unit mass,

10 $$a_{i}≡\frac{A_{i}}{W_{i}}$$

one obtains

11 $$Td_{i}S=\Delta S\;dT-\overset{N}{\underset{i=1}{\sum }}a_{i}\;dm_{i}$$

Taking the time derivative gives

12 $$\frac{d_{i}S}{dt}=\frac{\Delta S}{T}\frac{dT}{dt}-\overset{N}{\underset{i=1}{\sum }}\frac{a_{i}}{T}\frac{dm_{i}}{dt}$$

Finally, using the imposed heating rate $v=\frac{dT}{dt}$ , the entropy production rate can be written as

13 $$\frac{d_{i}S}{dt}=\frac{\Delta S}{T}v-\overset{N}{\underset{i=1}{\sum }}\frac{a_{i}}{T}\frac{dm_{i}}{dt}\geq 0$$

Therefore, the generalized flux–force pairs may be identified as

14 $$J_{T}=v,,X_{T}=\frac{\Delta S}{T}$$

which may be interpreted as a generalized thermal driving flux and its conjugate thermodynamic force associated with the externally imposed nonisothermal heating protocol and

15 $$J_{i}=\frac{{dm}_{i}}{dt},{,X}_{i}=-\frac{a_{i}}{T}$$

is the irreversible mass-evolution flux and its conjugate thermodynamic affinities. With these definitions, the entropy-production takes the standard bilinear form

16 $$\frac{d_{i}S}{dt}=J_{T}X_{T}+\overset{N}{\underset{i=1}{\sum }}J_{i}X_{i}\geq 0$$

The entropy-production expression obtained in eq 13 is particularly relevant in the context of irreversible thermodynamics because it explicitly incorporates the externally imposed heating rate as a thermodynamic driving contribution. Unlike conventional formulations of thermal degradation kinetics, where the heating rate enters only as an experimental control parameter, here the quantity $v=\frac{dT}{dt}$ appears directly as a generalized thermal flux coupled to the entropy production. This provides a nonequilibrium thermodynamic interpretation of nonisothermal TG experiments in which the thermal ramp actively drives the system away from equilibrium.

Within this framework, the degradation of the composite material is described as a driven irreversible process governed simultaneously by thermal forcing and chemical affinities. The resulting formulation establishes a direct connection between entropy production, mass-loss kinetics, and externally imposed thermal constraints, thereby extending the traditional Onsager-Prigogine formalism,^47–49^ to nonisothermal degradation processes under programmed heating conditions.

To the best of our knowledge, formulations that explicitly couple imposed heating ramps with TG mass-loss evolution within a Gibbs free-energy framework remain relatively unexplored in the literature. The present derivation follows the general irreversible thermodynamic methodology previously employed for mesoscopic driven systems and single-molecule dynamics^37^ here generalized to nonisothermal degradation processes under externally imposed heating ramps.

Starting from the bilinear form of the entropy-production, the generalized fluxes are related to their conjugate forces through linear phenomenological relations. In the present Gibbs free energy representation these coefficients should be understood as generalized phenomenological coefficients, and not as standard Onsager coefficients of the entropy representation. Thus, keeping the thermal flux and the mass fluxes defined through eq 14, 15 and 16, one may write

17 $$v=L_{TT}\frac{\Delta S}{T}-\overset{N}{\underset{j=1}{\sum }}L_{Tj}\frac{a_{j}}{T}$$

and

18 $$\frac{dm_{i}}{dt}=-L_{iT}\frac{\Delta S}{T}-\overset{N}{\underset{j=1}{\sum }}L_{ij}\frac{a_{j}}{T}$$

Solving eq 17 for the thermal force gives

19 $$\frac{\Delta S}{T}=\frac{v}{L_{TT}}+\overset{N}{\underset{j=1}{\sum }}\frac{L_{Tj}}{L_{TT}}\frac{a_{j}}{T}$$

Substitution of eq 19 into eq 18 yields

20 $$\frac{dm_{i}}{dt}=-\frac{L_{iT}}{L_{TT}}v-\overset{N}{\underset{j=1}{\sum }}\left[L_{ij}-\frac{L_{iT}L_{Tj}}{L_{TT}}\right]\frac{a_{j}}{T}$$

Equation 20 is the general mass-evolution equation obtained from the entropy-production formalism. The first term represents the direct coupling between the externally imposed heating ramp and the mass-loss rate of the *i*-th process, whereas the second term represents the relaxation driven by the chemical affinities. The terms with *j* ≠ *i* describe cross-couplings among the different irreversible degradation pathways.

In the weak-coupling approximation, the diagonal contribution dominates the phenomenological matrix. Therefore, the cross-couplings among different mass-loss processes (*j* ≠ *i*) are neglected and eq 20 reduces to

21 $$\frac{dm_{i}}{dt}=-L_{ii}\frac{a_{i}}{T}-\frac{L_{iT}}{L_{TT}}v$$

where we have introduced the effective phenomenological coefficients

22 $$L_{ii}≡L_{ii}-\frac{L_{iT}L_{Ti}}{L_{TT}}$$

These coefficients incorporate both the intrinsic irreversible response of the *i*-th degradation pathway and the correction induced by its coupling to the externally imposed thermal driving. The sign of the coupling term is chosen according to the convention that positive heating promotes mass loss. Thus, eq 21 constitutes the general evolution equation for the mass of each component under a nonisothermal TG experiment.

### Case of Biogenic Hydroxyapatite Composite

Bone powder is highly hydrophilic, meaning that it can readily absorb moisture from the environment. This absorbed water can be removed either by increasing the temperature or by storing the sample in a desiccator. This initial moisture loss process, denoted here as ϕ_0_, was not included in the model, since it does not constitute an intrinsic component of the bone ash itself but rather corresponds to physically adsorbed water.

Nevertheless, bone powder can be considered a composite material composed of at least four main components: water (1), with ϕ_1_ corresponding to its removal process; an organic phase (2, including fats and proteins, and 3, proteins that are resistant to higher temperatures, such as collagen), with ϕ_2_ and ϕ_3_ corresponding to the decomposition processes of components 2 and 3, respectively; and a mineral phase (4, biohydroxyapatite), with ϕ_4_ corresponding to its decomposition process.

The crystalline hydroxyapatite is embedded within an amorphous organic matrix, forming a continuous composite structure, as shown in Figure 2a. After the thermal treatment, only the BHA remains, and a porous structure is observed, as shown in Figure 2b. These free spaces arise from the evaporation and degradation of the organic components during the thermal process. The degradation process of bone powder during thermal treatment, illustrated in Figure 2c–g, can be understood as a continuous sequence of temperature-dependent transformations. At the initial stage (Figure 2c), the components preserve a defined structural arrangement. Water is primarily adsorbed on the surface of the hydroxyapatite crystals and on the collagen matrix. Although fat is not part of the mineralized portion of bone, small amounts may remain as lipid residues dispersed between the collagen fibers and hydroxyapatite crystals. At low temperatures (room temperature to 200 °C), the material loses both free water and loosely bound water associated with the organic matrix and mineral phase. Between 200 °C and 600 °C, the organic components, including fats, collagen, and other proteins, decompose, releasing volatile species and organic compounds^50^ (Figure 2e); this stage represents the major mass loss observed in the TG curve, and the progressive removal of organic matter allows the mineral phase to reorganize into a more ordered and crystalline structure (Figure 2f).^38^ At higher temperatures (600–900 °C), lattice-substituted carbonate ions undergo thermal decomposition, releasing CO_2_ and leading to additional mass loss, while promoting the formation of a more stoichiometric and thermally stable hydroxyapatite phase (Figure 2g). The observed crystallite growth at this stage suggests that mineral expansion is facilitated by the increasing free volume generated during the removal of organic matter and carbonates. Beyond these temperatures, the hydroxyapatite phase becomes highly stable, exhibiting minimal further mass loss.

**Figure 2:**
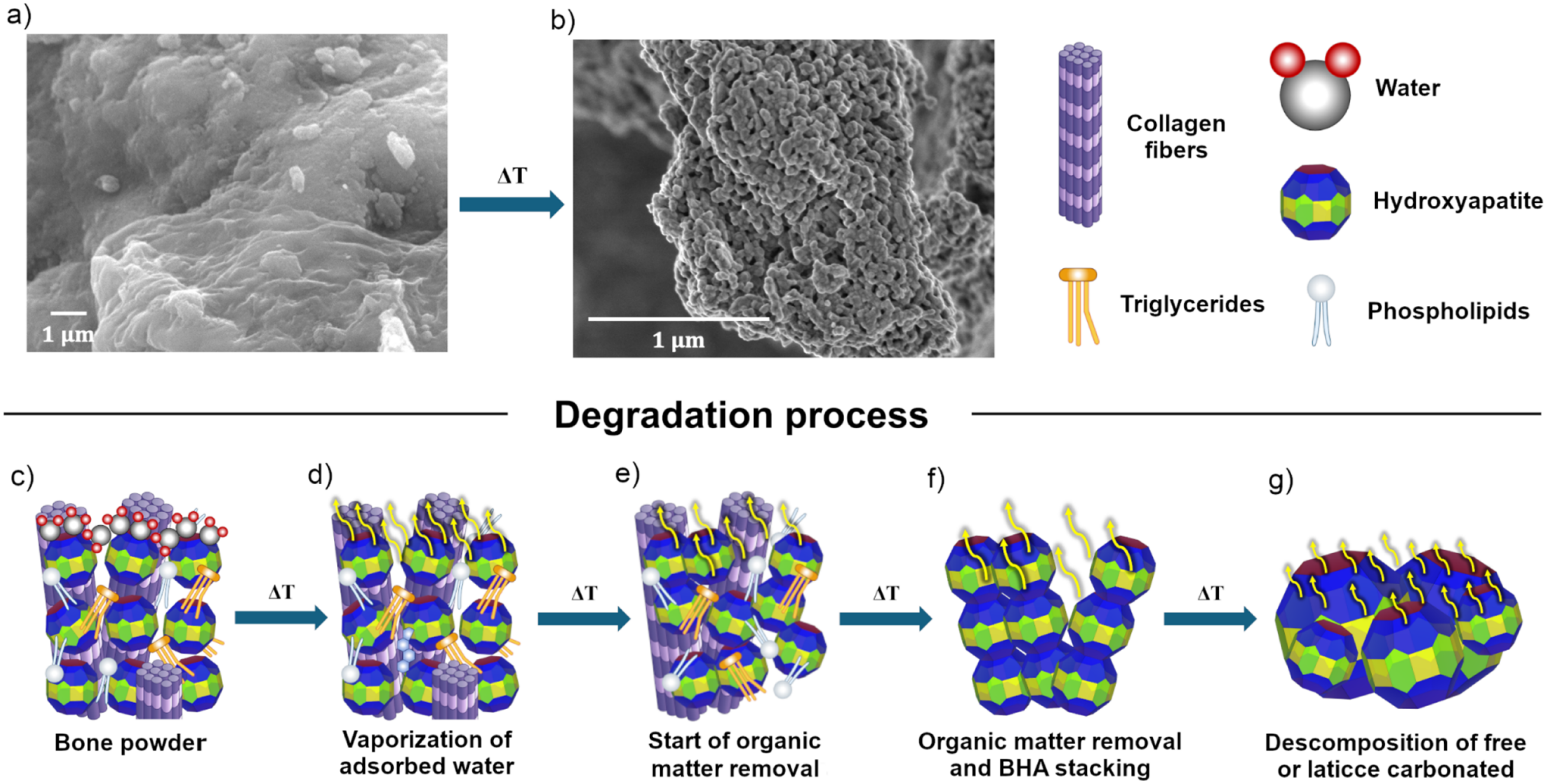
Schematic representation of the thermal transformation process of bone powder during heating. Panels (a) and (b) show SEM images of the initial bone powder and the resulting BHA after thermal treatment. Panels (c–g) illustrate the sequential stages of water evaporation, organic matter degradation, and carbonate decomposition as temperature increases. The scheme highlights the progressive removal of adsorbed water, lipids, and collagen, followed by the reorganization and crystal growth of the hydroxyapatite phase.

Based on the previously described thermal transformation processes, the evolution equations of the system are expressed as follows:

23a $$\frac{dm_{1}}{dt}=-L_{11}a_{1}-\frac{L_{1T}}{L_{TT}}v$$

23b $$\frac{dm_{2}}{dt}=-L_{22}a_{2}-\frac{L_{2T}}{L_{TT}}v$$

23c $$\frac{dm_{3}}{dt}=-L_{33}a_{3}-\frac{L_{3T}}{L_{TT}}v$$

23d $$\frac{dm_{4}}{dt}=-L_{44}a_{4}-\frac{L_{4T}}{L_{TT}}v$$

where *v* is defined in eq 3. In this set of equations, the first terms describe the relaxation driven by the corresponding chemical affinity, while the second terms account for the direct influence of the externally imposed heating ramp on the mass-loss dynamics. The experimental characterization of the composite sample reveals the existence of a temporal hierarchy (*τ*_1_ ≪ *τ*_2_ ≪ *τ*_3_ ≪ ⋯ ≪ *τN*). This hierarchy suggests that, in the simplest approximation, each chemical affinity takes the form

24 $$a_{i}=RT\;\ln\left|\frac{m_{i}}{m_{i0}}\right|+a_{0}$$

where *m_i0_* is the initial mass of the *i*-th compound and *a*_0_ is a reference value. Approximating the logarithm terms about the unity up to first order, and canceling constant terms allows for the simpler analytical form

25 $$a_{i}≃RT\left(\frac{m_{i}-m_{i0}}{m_{i0}}\right)$$

Substituting eq 28 into the corresponding terms of eq 23, a final set of differential equations is obtained, which describes the mass evolution of the composite in the TGA. This final set of equations can be written in the compact form

26a $$\frac{dm_{1}}{dt}=-\frac{1}{\tau_{1}}\left[\left(m_{1}-m_{10}\right)+v\beta_{1}\right]$$

26b $$\frac{dm_{2}}{dt}=-\frac{1}{\tau_{2}}\left[\left(m_{2}-m_{20}\right)+v\beta_{2}\right]$$

26c $$\frac{dm_{3}}{dt}=-\frac{1}{\tau_{3}}\left[\left(m_{3}-m_{30}\right)+v\beta_{3}\right]$$

26d $$\frac{dm_{4}}{dt}=-\frac{1}{\tau_{4}}\left[\left(m_{4}-m_{40}\right)+v\beta_{4}\right]$$

Here, to simplify the notation, phenomenological heating rates have been introduced *β_i_* *τ_i_* (*L_iT_*/*L_TT_*) and the inverse characteristic relaxation times are defined by $\tau_{i}^{-1}≡L_{ii}\left(RT/m_{i0}\right)$ for *i* = 1, 2, 3, 4.

As mentioned above, the phenomenological parameters (β_*i*_) entering the evolution equations should not be interpreted as strict Onsager coefficients in the classical near-equilibrium sense, but rather as generalized ratios between phenomenological coefficients governing the coupling between thermal forcing and mass evolution under nonisothermal conditions. Physically, the quantities (β_*i*_) may be interpreted as effective mobility factors that characterize the ability of each irreversible evaporation or degradation mechanism to respond to the externally imposed heating ramp $\left(v=\frac{dT}{dt}\right)$ . Moreover, the products (β_*i*_v) determine the average contribution of each irreversible process to the slope of the mass-evolution curves, thus directly controlling the effective rate of mass loss observed in the TG experiments. Consequently, the parameters (β_*i*_) may be interpreted as effective thermal-to-mass mobility factors describing how efficiently the externally imposed thermal forcing is converted into irreversible mass evolution.

Processes dominated by rapid surface evaporation or weakly hindered desorption are expected to exhibit comparatively large effective mobilities and weaker sensitivities to the thermal ramp, leading to small variations of the apparent enthalpies with *v*. In contrast, processes controlled by slower irreversible mechanisms, such as internal diffusion, transport through porous regions, structural rearrangements, or cooperative dehydration dynamics, may display smaller effective mobilities and stronger dependence on the imposed heating conditions. Consequently, variations in the functions $H_{i}\left(v\right)$ and in their derivatives, $\frac{d\Delta H_{i}}{dv}$ , may be interpreted as indirect signatures of changes in the effective irreversible mobilities governing the degradation dynamics. In particular, pronounced slope changes or extrema in the apparent enthalpy curves suggest crossovers between competing transport, diffusion, evaporation, or desorption mechanisms activated under different thermal driving conditions.

Within the framework of irreversible thermodynamics, no explicit functional form is prescribed for the phenomenological coefficients $L_{ii}$ . Therefore, it is necessary to resort to kinetic theories, such as the Eyring–Polanyi formalism,^51–53^ which enable the identification of these coefficients with elementary rate processes and establish a direct connection with Arrhenius-type relations. In this context, the inverse relaxation times can be interpreted in terms of effective evaporation or degradation rates associated with each component (eq 33).

The inverse relaxation time can be expressed in terms of the well-established Eyring–Polanyi formulation for evaporation rates, namely

27 $$k_{i}^{\left(ev\right)}≡\tau_{i}^{-1}=k_{0,i}^{\left(ev\right)}e^{-\frac{\Delta H_{i}}{RT}}$$

where, $k_{0,i}^{\left(ev\right)}$ is the pre-exponential factor and Δ*H*_*i*_ corresponds to the evaporation enthalpy of the *i*-th component. This formulation provides a physically consistent framework to relate the macroscopic mass loss to the underlying thermally activated processes. In particular, the pre-exponential factor $k_{0,i}^{\left(ev\right)}$ represents a characteristic attempt frequency, while the apparent evaporation enthalpy Δ*H*_*i*_ governs the temperature dependence of the relaxation time scales.

The set of eq 26 must be solved simultaneously to determine the temperature evolution of the total mass in terms of temperature *M*(*T*) = *m*_1_(*T*)+*m*_2_(*T*)+*m*_3_(*T*)+*m*_4_(*T*). This is achieved by utilizing the equivalence *dt* = *v* ^–1^ *dT*, which follows directly from eq 3.

## Results and Discussion

Figure 3a displays the TG curves of the bone powder sample obtained at different heating rates (*v*): 3, 5, 7, 25, 50, 75, and 100 °C min^–1^. Figure 3b shows the corresponding DDTG. Each peak in the DDTG profile represents a rapid change in the mass-loss rate, indicating a major thermal event, such as a decomposition peak or gas release associated with a specific component (ϕ_*i*_). For this composite material (bone powder), the overall mass loss can be attributed to several processes: the removal of environmental water (ϕ_0_(*v*)), the release of bound water (ϕ_1_(*T,v*)), the degradation of the organic matrix (ϕ_2_(*v*) and ϕ_3_(*v*)), and the decomposition of the inorganic matrix (ϕ_4_(*v*)) (Figure 2). The pronounced shift of the curves toward higher temperatures is directly attributable to the increase in heating rate. This effect becomes even more evident in the displacement of the maxima of the DDTG for each thermogram, as presented in Figure 3b.

**Figure 3:**
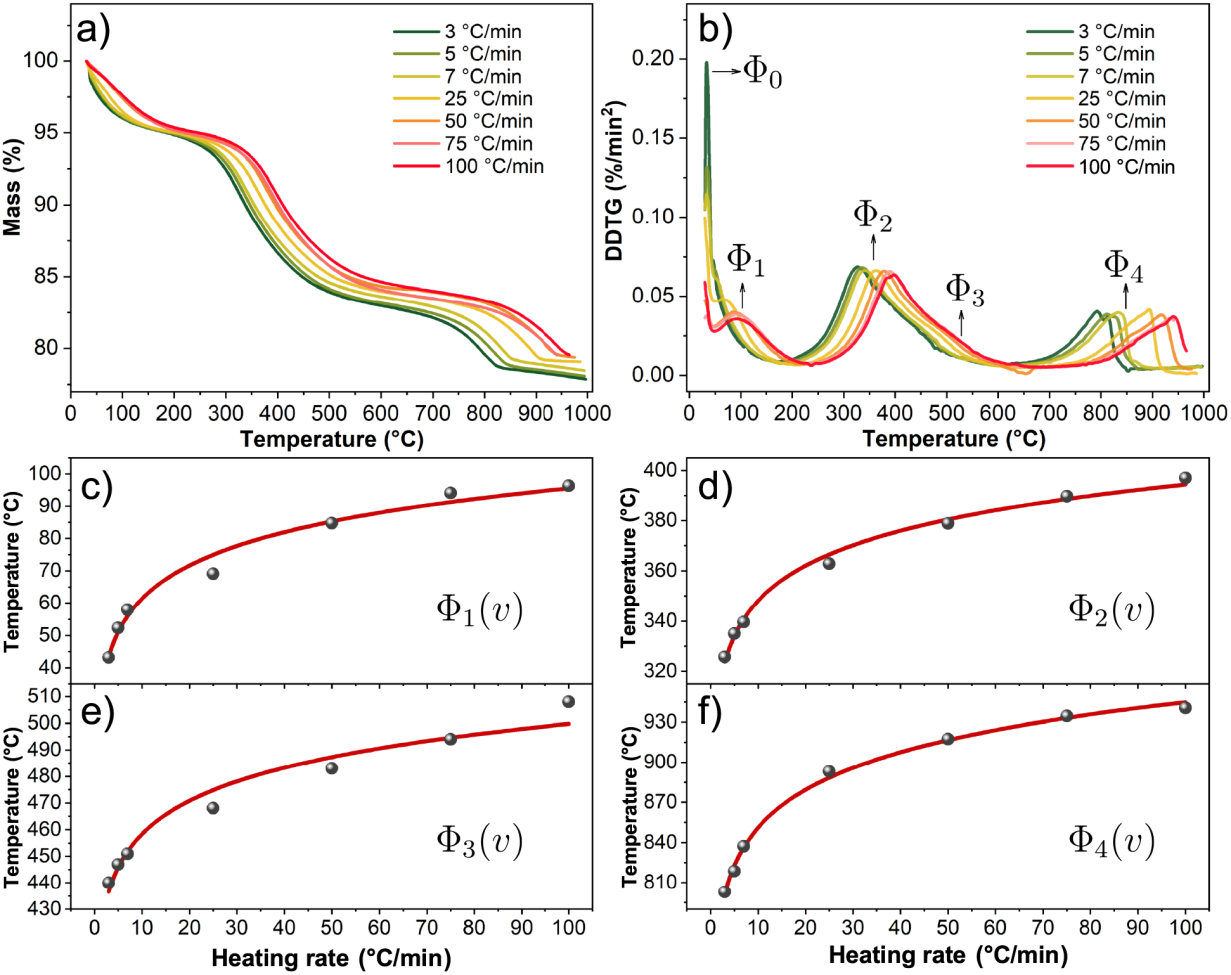
(a) TG curves and (b) second-derivative thermogravimetric (DDTG) profiles for the BHA composite at heating rates of 3, 5, 7, 25, 50, 75, and 100 ^◦^C/min. Panels (c–f) illustrate the evolution of the maximum peak positions for each characteristic degradation stage (ϕ_0_, ϕ_1_, ϕ_2_, ϕ_3_, and ϕ_4_) as a function of the heating rate.

The trend observed for each degradation event indicates that the peak position shifts monotonically to higher temperatures as the heating rate increases. Figures 3c–f illustrate this effect, showing how the maximum of each DDTG peak moves as a function of the heating rate. This shift is primarily driven by kinetic limitations and heat transfer effects. At higher heating rates, the sample spends less time at each temperature, preventing the evaporation or decomposition reaction from reaching the same extent before the temperature continues to rise. To compensate for this shorter reaction time, the system requires a higher temperature to achieve the same degree of mass loss, since reaction rates increase exponentially with temperature. Additionally, at elevated heating rates, the temperature distribution within the sample becomes less uniform due to limited heat diffusion, delaying thermal transfer toward the material’s core and resulting in nonhomogeneous degradation.

To quantitatively reproduce these experimental trends and extract the kinetic parameters of the model in eq 33 (*i.e. m*_*i*_, β_*i*_, τ_*i*_, Δ*H*_*i*_), the system of equations defined in eqs 23–26 is solved numerically using Wolfram Mathematica (Supporting Information Code S1 and Figure S1). The parameter set is obtained through an optimization procedure in which the values of the kinetic and thermophysical variables are iteratively adjusted so that their combination yields the best possible agreement with the experimental TG and DDTG curves (Supporting Information Tables S1–S3). This fitting routine minimizes the discrepancy between measured and simulated mass loss profiles across all heating rates, ensuring that the model accurately captures both the peak positions and the overall shape of each degradation event. The optimized parameters used in this study are reported in the Supporting Information.

Figure 4 shows the fitting of the TG curves with an error below 0.05%. The lowest coefficient of determination, *R* ^2^ = 0.9989, is found for the sample heated at 50 ^◦^C/min (Figure 4e). This lower value arises because the TGA curve is incomplete, as the instrument loses sensitivity in this temperature range due to the high heating rate. The same effect is observed in Figure 4f and 4g, in addition to the fact that the final transition (ϕ_4_), associated with the loss of carbonate ions, shifts to such high temperatures that it does not fully develop even at 1000 ^◦^C, as seen in the DDTG curve (Figure 3b). This implies that even though the heating rate is high, the model continues to predict the degradation behavior of bone powder correctly.

**Figure 4:**
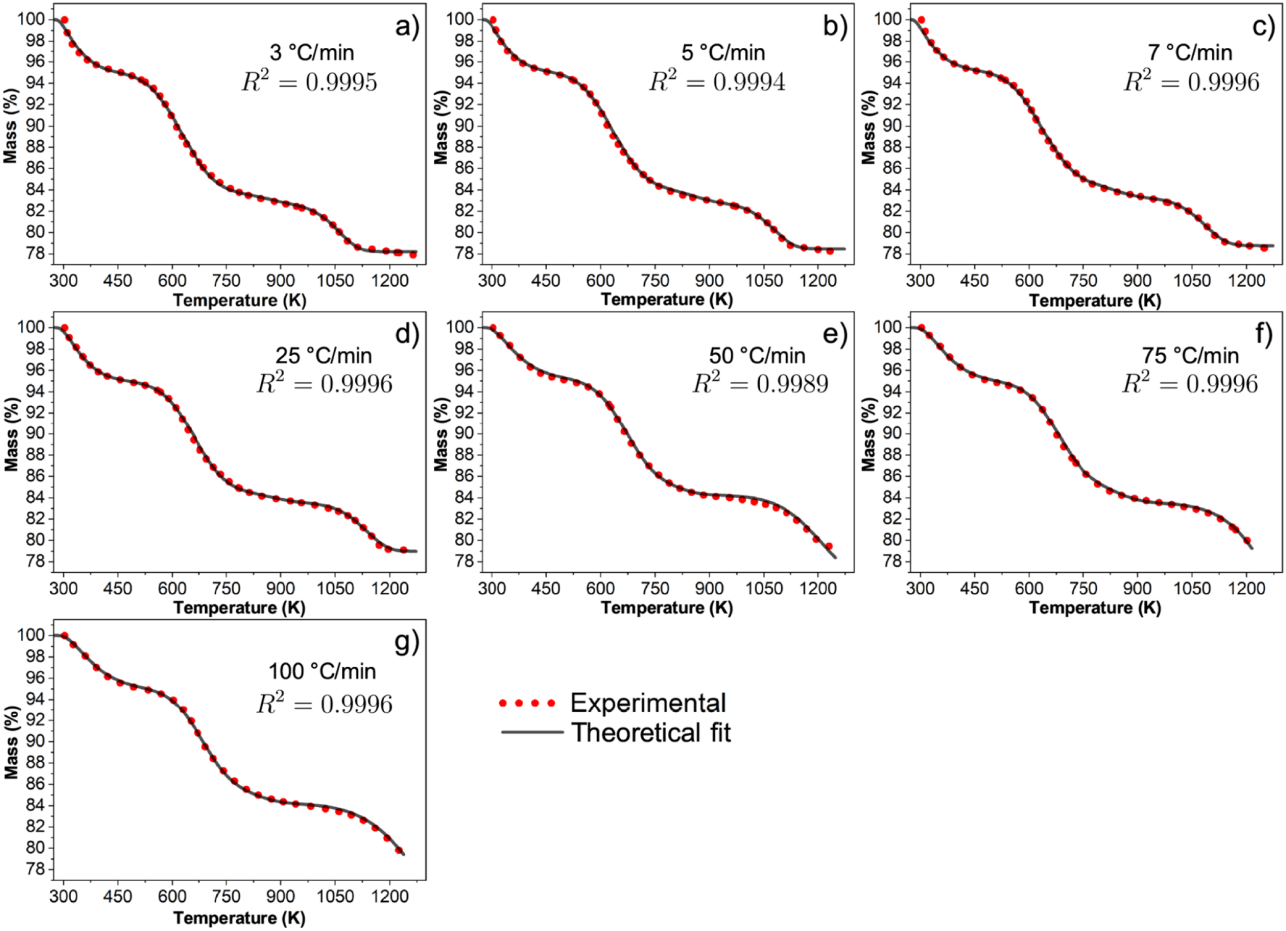
Comparison between the experimental data and the results obtained from the kinetic model for heating rates of (a) 3, (b) 5, (c) 7, (d) 25, (e) 50, (f) 75, and (g) 100 °C/min. In each panel, the red dashed line represents the experimental measurements, whereas the solid black line corresponds to the best fit obtained from the kinetic model through the numerical solution of the set of eq 26. The coefficient of determination (*R* ^2^), displayed in each panel, is included as a measure of the goodness of fit between the experimental data and the fitted model results.

As a result of the curve fitting process of the experimental data, the enthalpies of evaporation (Δ*H*_*i*_) of the composite components as a function of the heating rate were obtained, as shown in Figure 5. To analyze the trends shown in this figure, we focus on the heating-rate sensitivity of the apparent enthalpies, quantified by $\frac{d\Delta H_{i}}{dv}$ . This derivative provides a direct measure of how strongly each fitted enthalpy depends on the externally imposed thermal ramp. In the low-ramp interval *v* = 3, 5, and 7^◦^C/min, the apparent enthalpies vary weakly with *v*, so that $\frac{d\Delta H_{i}}{dv}\approx 0$ within the resolution of the fitting procedure. This condition defines the quasi-stationary low-ramp regime highlighted in red in Figure 5. Within this interval, the fitted apparent enthalpies are expected to approach the intrinsic enthalpic contributions associated with each dominant mass-loss stage. Outside this interval, the slopes of the Δ*H*_i_(*v*) curves become appreciably different from zero, indicating that the extracted apparent enthalpies become progressively perturbed by the externally imposed heating protocol.^54^ This behavior is analogous to the well-known dependence of apparent activation energies on heating conditions in nonisothermal kinetic analyses.^42,54,55^ Positive values of $\frac{d\Delta H_{i}}{dv}$ indicate increasing kinetic resistance under faster heating, whereas negative values suggest the activation of transport-limited or alternative degradation pathways. Therefore, slope changes or extrema in Δ*H*_i_(*v*) are interpreted as kinetic crossovers between dominant irreversible mechanisms, rather than as equilibrium phase transitions. The region of Δ*H*_i_ values obtained at low heating rates, highlighted in red in Figure 5, indicates that low heating rates are the most suitable for sintering bone powder to obtain BHA while maintaining a quasi-static process. This occurs because, at higher heating rates, the process is influenced not only by the sintering temperature but also by the heating rate itself, which becomes a critical factor altering the system’s thermal response. Under these conditions, local thermodynamic quasi-equilibrium conditions can no longer be approximately maintained, and additional deviations arise from instrumental limitations, such as measurement delays.

**Figure 5:**
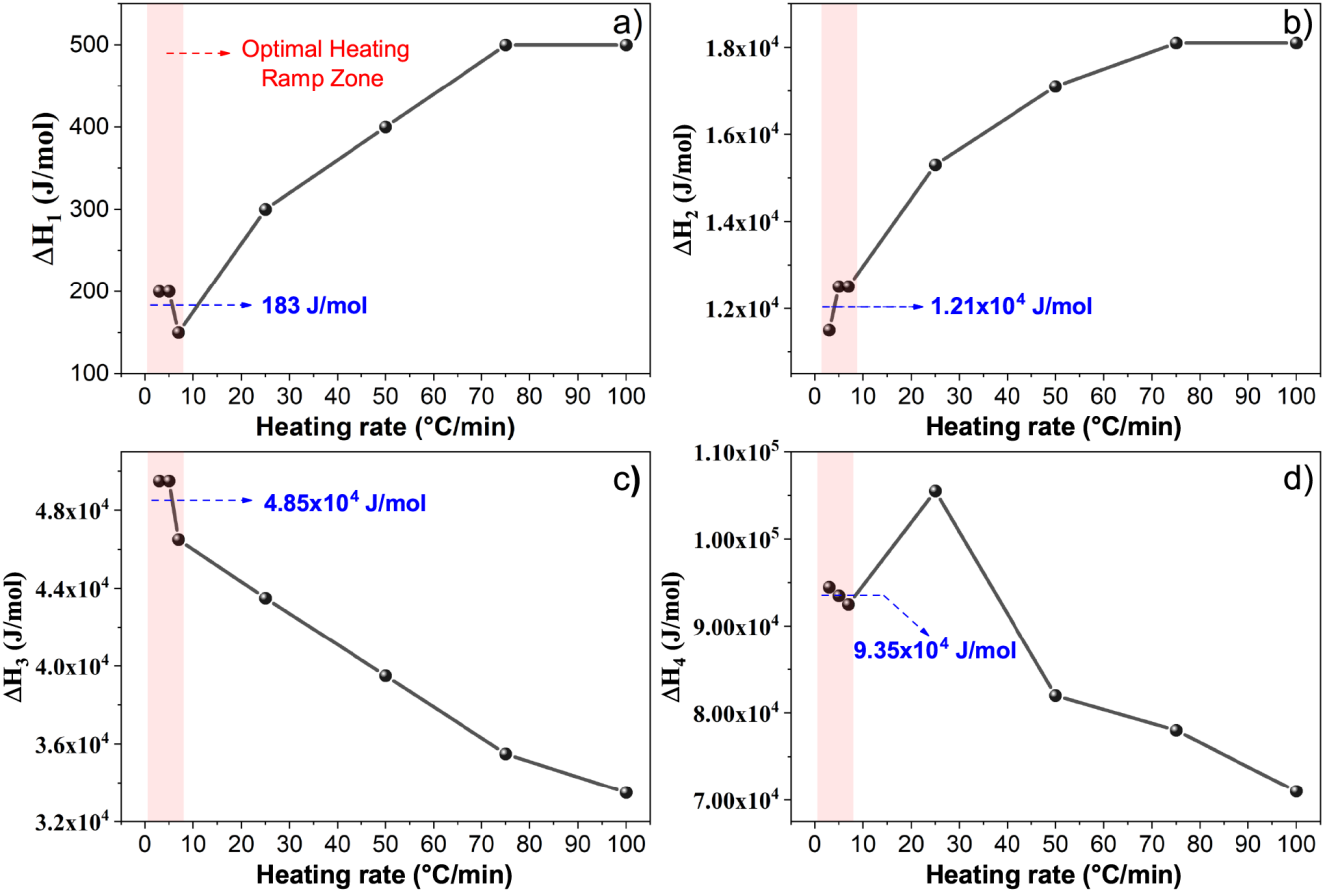
Apparent evaporation enthalpy of (a) bound water, (b) organic matrix, (c) surface carbonate ions, and (d) HA. The shaded red region indicates the low-heating-rate quasi-stationary regime for which the apparent enthalpies satisfy approximately (*d*Δ*H_i_*/*dv* ≈ 0), implying weak dependence of the fitting parameters on the externally imposed thermal ramp.

The enthalpies Δ*H*_1_ and Δ*H*_2_ exhibit a monotonic increase as a function of the heating rate. This trend indicates that these processes remain predominantly governed by Arrhenius-type chemical kinetics, in which the thermal delay introduced at higher heating rates is interpreted by the model as a stronger temperature dependence, thereby yielding larger apparent enthalpies. This increase can also be understood in terms of the physical location of the volatile species: components that evaporate from regions closer to the surface experience weaker diffusion limitations, making their evaporation more sensitive to the external temperature ramp. As discussed above, when the heating rate becomes an additional variable in the system, thermal diffusion within the sample is no longer homogeneous, enhancing the kinetic contribution to the observed Δ*H*_*i*_. In contrast, the enthalpies Δ*H*_3_ and Δ*H*_4_ exhibit a decreasing trend as the heating rate increases. This behavior reflects a transition from an Arrhenius-controlled regime to a diffusion-dominated regime. At this stage of the process, the material is no longer a continuous solid: the extensive removal of water and organic matter results in a porous microstructure, making heat transfer increasingly inefficient. The reduction in effective thermal conductivity leads to pronounced temperature gradients within the solid, limiting the evaporation of the remaining volatile species not by the intrinsic activation barrier but by the rate at which heat can be transported through the porous matrix. Furthermore, partial sintering and local softening of the mineral grains modify the pathways through which volatiles escape, creating constricted diffusion channels and altering the energy required for their release. Under these conditions, the kinetic model interprets the weakened temperature dependence as a lower apparent enthalpy, since the process becomes governed by mass and heat transport limitations rather than by a purely thermally activated mechanism. As a result, Δ*H*_3_ and Δ*H*_4_ decrease with increasing heating rate, reflecting the progressive dominance of physical transport constraints over Arrhenius-type chemical kinetics.

The low-heating-rate regime characterized by $\left(\frac{d\Delta H_{i}}{dv}\approx 0\right)$ is also associated with a weak dependence of the phenomenological parameters (β_*i*_) on the imposed thermal ramp. This behavior suggests that the characteristic relaxation dynamics becomes quasi-stationary in this interval, allowing the extracted apparent enthalpies and kinetic parameters to approach their intrinsic values.

Therefore, the first three heating rates exhibit approximately constant Δ*H*_*i*_ values, as shown in Table 1. Considering the mean value marked in blue in Figure 5, the quasi-stationary low-ramp or reduced-sensitivity regime is represented by 5 °C/min, as it is the closest to that mean.

**Table 1:** Apparent Evaporation Enthalpy of Each Component of Bone Powder for Each Heating Rate

Heating rate (^◦^C/min)	Δ*H*_1_ (kJ/mol)	Δ*H*_2_ (kJ/mol)	Δ*H*_3_ (kJ/mol)	Δ*H*_4_ (kJ/mol)
3	0.20	11.50	49.50	94.50
5	0.20	12.50	49.50	93.50
7	0.15	12.50	46.50	92.50
25	0.30	15.30	43.50	105.50
50	0.40	17.10	39.50	82.00
75	0.50	18.10	35.50	78.00
100	0.50	18.10	33.50	71.00

## Conclusions

A thermokinetic model grounded in nonequilibrium thermodynamics was developed to describe the thermal degradation of multicomponent composite materials, using a bone-derived composite as a representative case study. Starting from the Gibbs free-energy balance and the entropy-production formalism, and employing generalized phenomenological relations between thermodynamic fluxes and forces, a set of evolution equations was derived to link mass-loss kinetics to their corresponding thermodynamic driving forces. By incorporating a temperature-dependent chemical potential and explicitly including the externally imposed heating rate as an input parameter, a physically consistent system of differential equations (eq 26) was obtained to describe the temperature-dependent mass evolution of an N-component composite under a nonisothermal TG experiment. The model was solved numerically and validated against experimental TG curves of bovine bone powder at seven different heating rates. The agreement between the theoretical predictions and experimental measurements was excellent, with errors below 0.05% and coefficients of determination exceeding *R* ^2^ = 0.998. These results demonstrate that the proposed framework accurately reproduces both the overall mass-loss behavior and the detailed kinetic structure of each degradation event. The model achieves this high predictive capability while relying solely on physically meaningful parameters: relaxation times, heating rate, and evaporation enthalpies, without introducing unnecessary degrees of freedom.

The evaporation enthalpies extracted from the model correspond to apparent enthalpies, as they depend not only on intrinsic interactions but also on heat and mass transfer, as well as on limitations associated with the evolving microstructure during thermal decomposition. At low heating rates, where the system behaves quasi-statically, the extracted enthalpies approximate the intrinsic evaporation energies of the composite’s constituents. As the heating rate increases, thermal gradients, decreased thermal conductivity resulting from pore formation, and partial sintering of mineral grains introduce diffusion-dominated behavior. Under these conditions, the apparent enthalpies increase or decrease, depending on whether the corresponding component evaporates from near-surface regions (kinetically controlled) or from restricted diffusion pathways formed after organic matter removal (transport-limited). This analysis confirms that the thermal response is strongly constrained by diffusive phenomena at elevated heating rates.

The identification of a stable enthalpy region at low heating rates offers a practical criterion for selecting appropriate experimental conditions to obtain reliable evaporation enthalpies. For the BHA, a heating rate of 5 ^◦^C/min was identified as belonging to the quasi-stationary low-ramp or reduced-sensitivity regime, which helps ensure local thermodynamic quasi-equilibrium conditions during measurement. This result provides a rational basis for determining suitable sintering ramps, especially when preserving carbonate content, crystallite size, and phase purity is required. Beyond the specific application to bone-derived hydroxyapatite, the proposed thermokinetic framework is general and applicable to composite materials with an arbitrary number of components. By establishing a deterministic link between TG measurements and underlying physicochemical interactions, such as intermolecular energies, diffusion barriers, and heat-transport limitations, the model extends the interpretation of TGA data beyond empirical mass-loss curves and into the domain of rigorous thermodynamic and physicochemical analysis. This theoretical foundation provides valuable insights for optimizing thermal processing conditions and for predicting the functional evolution of composite materials in a wide range of scientific and engineering applications.

## Supplementary Material



## Notes

Additional values of the different variables for each fitting (PDF)
