# Supplementary material for: Thermo-Kinetic Framework for TGA Curve Modeling and Evaporation Enthalpy Determination in Composite Materials: The Case of Bone-Derived Hydroxyapatite
Source: J Phys Chem B. 2026 Jun 24;130(27):6826–37. doi: 10.1021/acs.jpcb.6c01424 (PMC13359376; doi:10.1021/acs.jpcb.6c01424)
Supplement: Supplementary file 1 [file jp6c01424_si_001.pdf]

Supporting Information

Thermo-kinetic framework for TGA curve modeling and evaporation enthalpy determination in composite materials: the case of bone-derived hydroxyapatite

Leon R. Bernal-Alvarez*,† (*ORCID: 0009-0006-3232-7368*), Ivan Santamaría-Holek‡ (*ORCID: 0000-0002-5306-197X*), Jose Luis Rivera-Armenta¶ (*ORCID: 0000-0002-9076-2353*), and Cristian F. Ramirez-Gutierrez*,§,‖ (*ORCID: 0000-0002-0450-5810*)

†*Universidad Nacional Autónoma de México, Centro de Física Aplicada y Tecnología Avanzada, Campus Juriquilla 76230, Qro., Mexico*

‡*UMDI-Facultad de Ciencias, Universidad Nacional Autónoma de México Campus Juriquilla 76230, Qro., Mexico*

¶*Tecnológico Nacional de México/Instituto Tecnológico de Ciudad Madero, Centro de Investigación en Petroquímica, Prol Bahía de Aldahir y Av. de las Bhabias S/N Parque de la pequeña y mediana industria, 89600, Altamira, Tmas., Mexico*

§*Department of Ecology, Environment and Geoscience, Umeå Universitet, 901 87 Umeå, Sweden*

#*I.S.H contributed equally as cofirst authors*

**Corresponding authors: leonbernal@pceim.unam.mx (Leon R. Bernal-Alvarez); cristian.ramirez.gutierrez@umu.se (Cristian F. Ramirez-Gutierrez)*

Code S1 describes the implementation of the thermodynamic Arrhenius-based model used to fit the experimental thermogravimetric analysis (TGA) data in Wolfram Mathematica with *Site License (Universidad Nacional Autonoma de Mexico)*. The procedure begins with importing the experimental dataset containing temperature and mass percentage values. Once the data are loaded, the experimental thermogravimetric (TG) curve is plotted to visualize the degradation behavior and identify the different thermal decomposition regions.

Subsequently, the model's initial parameters are introduced. These include the total mass (M), the mass fractions associated with each degradation stage ($m10$, $m20$, $m30$, and $m40$), the heating rate (v), kinetic parameters as slope-related parameters (bw, bop, bca, and bbh) and characteristic degradation times (tw, top, tca, and tbh), apparent enthalpy evaporation ($H1$, $H2$, $H3$, and $H4$), and the gas constant ($R = 8.314\ \mathrm{J/(mol \cdot K)}$). The fitting procedure is performed sequentially to improve convergence and physical consistency. First, the inflection points of the experimental curve are identified to estimate the mass contributions associated with each degradation event. Then, the characteristic degradation times and apparent

enthalpy of evaporation are adjusted to reproduce the onset and end regions of each decomposition process. Finally, the slope-related parameters are refined to accurately reproduce the degradation rates and the curvature of the experimental signal.
After defining the parameters, the coupled system of differential equations is solved numerically using NDSolve. The model considers four independent degradation processes governed by Arrhenius-type kinetics. Each process contributes individually to the total mass loss during heating.

Once the numerical solution is obtained, the individual degradation curves and the total predicted mass-loss curve are plotted separately (see Figure S1). This step allows verification of the contribution of each degradation mechanism and facilitates independent evaluation of the fitting quality for each thermal event. Afterward, the total model prediction, corresponding to the sum of all degradation contributions, is superimposed on the experimental TGA data to evaluate the overall fitting accuracy.

Additionally, the coefficient of determination (R^2) is calculated to quantitatively evaluate the goodness of fit between the experimental data and the thermodynamic model. Values of R^2 close to 1 indicates excellent agreement between the experimental and simulated curves.

Code S1. Code of the thermodynamic model to adjust to the experimental TGA data. Using Wolfram Mathematica with *Site License (Universidad Nacional Autonoma de Mexico)*.

```
(* :::::::::::::::::::::::::::::::::::::::::::::::::::::::::::::::::::::::::::::::::::::::::::::::::: *)
(* Code S1. Thermodynamic Arrhenius model for TGA fitting          *)
(* Wolfram Mathematica - Site License (UNAM)                        *)
(* :::::::::::::::::::::::::::::::::::::::::::::::::::::::::::::::::::::::::::::::::::::::::::::::::: *)

(* ===================== *)
(* 1. IMPORT EXPERIMENTAL DATA *)
(* ===================== *)

(* Import the experimental TGA dataset *)
AdP = Import["(* Enter the file path *)"];

(* Display imported data *)
AdP;

(* ===================== *)
(* 2. PLOT EXPERIMENTAL DATA *)
(* ===================== *)

(* Plot experimental TGA curve *)
FigTGAexp01ADP =
 ListPlot[
```

```
  AdP,
  Joined -> True,
  PlotStyle -> Black,
  Frame -> True,
  FrameLabel -> {
   Style["Temperature [K]", 14],
   Style["Mass percentage [%]", 14]
  },
  ImageSize -> 500
 ];

(* ===================== *)
(* 3. DEFINE MODEL PARAMETERS *)
(* ===================== *)

(* Clear all previous variables *)
Clear[
 tmax, M, m10, m20, m30, m40,
 v, bw, bop, bca, bbh,
 tw, top, tca, tbh,
 H1, H2, H3, H4, R
];

(* Maximum temperature *)
tmax = (* Value in Kelvin *);

(* Initial masses associated with each degradation stage *)
{
 M = (* Total mass value *),
 m10 = (* Stage 1 mass *),
 m20 = (* Stage 2 mass *),
 m30 = (* Stage 3 mass *)
};

(* Remaining mass *)
m40 = M - m10 - m20 - m30;

(* Heating rate *)
v = (* Heating rate value *);

(* Slope correction parameters *)
{
 bw = (* Value *),
```

```
 bop = (* Value *),
 bca = (* Value *),
 bbh = (* Value *)
};

(* Characteristic degradation times *)
{
 tw = (* Value *),
 top = (* Value *),
 tca = (* Value *),
 tbh = (* Value *)
};

(* Activation energies and gas constant *)
{
 H1 = (* Value *),
 H2 = (* Value *),
 H3 = (* Value *),
 H4 = (* Value *),
 R = 8.314 (* Gas constant *)
};

(* ===================== *)
(* 4. SOLVE DIFFERENTIAL EQUATIONS *)
(* ===================== *)

(* Numerical solution of the Arrhenius degradation model *)
Casol2exponencial =
 NDSolve[
  {
   T'[t] == v,

   (* Degradation process 1 *)
   m1'[t] ==
    -(1/tw) Exp[-(H1/(R*T[t]))]
    ((m1[t] - m10) + bw),

   (* Degradation process 2 *)
   m2'[t] ==
    -(1/top) Exp[-(H2/(R*T[t]))]
    ((m2[t] - m20) + bop),

   (* Degradation process 3 *)
```

```
    m3'[t] ==
     -(1/tca) Exp[-(H3/(R*T[t]))]
     ((m3[t] - m30) + bca),

    (* Degradation process 4 *)
    m4'[t] ==
     -(1/tbh) Exp[-(H4/(R*T[t]))]
     ((m4[t] - m40) + bbh),

    (* Initial conditions *)
    T[0] == 1,
    m1[0] == m10,
    m2[0] == m20,
    m3[0] == m30,
    m4[0] == m40
   },

   (* Variables *)
   {T, m1, m2, m3, m4},

   (* Time interval *)
   {t, 0.0, tmax}
  ];

(* ==================== *)
(* 5. PLOT INDIVIDUAL DEGRADATION CONTRIBUTIONS *)
(* ==================== *)

Camodeloarrheniusv =
 Plot[
  Evaluate[
   {
    (* Total model *)
    (m1[(t - 273.15)/v]/M)*100 +
    (m2[(t - 273.15)/v]/M)*100 +
    (m3[(t - 273.15)/v]/M)*100 +
    (m4[(t - 273.15)/v]/M)*100,

    (* Individual contributions *)
    (m1[(t - 273.15)/v]/M)*100,
    (m2[(t - 273.15)/v]/M)*100,
    (m3[(t - 273.15)/v]/M)*100,
    (m4[(t - 273.15)/v]/M)*100
```

```
  } /. Casol2exponencial
 ],

 {t, 273.15, tmax},

 PlotStyle -> {
  {Black, Dashed},
  Red,
  Blue,
  Green,
  Orange
 },

 Frame -> True,
 Axes -> False,
 PlotRange -> All,

 FrameLabel -> {
  Style["T [K]", 14],
  Style["Total mass [%]", 14]
 },

 ImageSize -> 500
];

(* ===================== *)
(* 6. SUPERIMPOSE MODEL AND EXPERIMENTAL DATA *)
(* ===================== *)

(* Compare experimental TGA data with fitted model *)
Show[
 FigTGAexp01ADP,
 Camodeloarrheniusv
]

(* ===================== *)
(* 7. CALCULATE R^2 COEFFICIENT *)
(* ===================== *)

(* Experimental temperatures *)
TempExp = AdP[[All, 1]];
```

```
(* Experimental mass percentages *)
MassExp = AdP[[All, 2]];

(* Evaluate model at experimental temperatures *)
MassModel =
 Table[
  Evaluate[
   (
    (m1[(T - 273.15)/v]/M)*100 +
    (m2[(T - 273.15)/v]/M)*100 +
    (m3[(T - 273.15)/v]/M)*100 +
    (m4[(T - 273.15)/v]/M)*100
   ) /. Casol2exponencial
  ],
  {T, TempExp}
 ];

(* Residual sum of squares *)
SSres =
 Total[(MassExp - MassModel)^2];

(* Total sum of squares *)
SStot =
 Total[(MassExp - Mean[MassExp])^2];

(* Coefficient of determination *)
R2 = 1 - (SSres/SStot);

(* Display R^2 *)
Print["R^2 = ", R2];
```

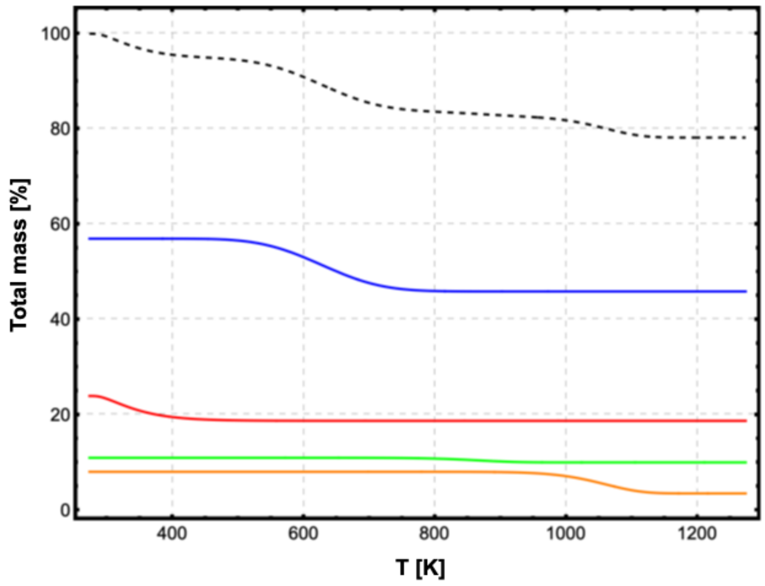

Figure S1. Comparison between the experimental TG curve and the individual degradation contributions obtained from the Arrhenius-based thermodynamic model implemented in Wolfram Mathematica. The dashed black curve corresponds to the experimental TGA data, while the colored solid curves represent the individual degradation processes obtained from the numerical solution of the coupled differential equation system (red: $m10$; blue: $m20$; green: $m30$; orange: $m40$). Each contribution is associated with a specific thermal decomposition event defined by its mass fraction, characteristic degradation time, activation energy, and slope-adjustment parameter. The sum of all modeled contributions reproduces the sample's overall thermal degradation behavior.

Table S1. Masses of composite constituents in TGA modeling.

Heating rate (°C/min)	M_{Total} (mg)	m_{10} (mg)	m_{20} (mg)	m_{30} (mg)	m_{40} (mg)
3	11.37190	11.17289	10.32454	9.69454	9.03952
5	11.46406	11.23592	10.40248	9.77998	9.13914
7	11.05006	10.81027	10.05776	9.46990	8.81905
25	11.27321	11.02745	10.24509	9.69383	8.98925
50	12.61088	12.43811	11.48472	10.87562	10.12779
75	13.89182	13.58203	12.60543	11.93446	11.08289
100	11.43504	11.17660	10.38759	9.84099	9.174332

Table S2. Phenomenological heating ramps.

Heating rate (°C/min)	β_1 (mg)	β_2 (mg)	β_3 (mg)	β_4 (mg)
3	0.59250	1.26000	0.11000	0.85000
5	0.59250	1.24000	0.15000	0.65000
7	0.55250	1.14000	0.15000	1.16000
25	0.58250	1.14000	0.13000	0.95000
50	0.62525	1.18000	0.18000	0.10000
75	0.72525	1.23000	0.33000	1.90000
100	0.58525	1.00000	0.22000	1.90000

Table S3. Characteristic relaxation times.

Heating rate (°C/min)	τ_1 (s)	τ_2 (s)	τ_3 (s)	τ_4 (s)
3	13.225500	0.620000	0.000900	0.000010
5	8.225500	0.280000	0.000450	0.000010
7	6.225500	0.200000	0.000450	0.000010
25	1.525000	0.030000	0.000450	0.000001
50	0.925500	0.009000	0.000200	0.000050
75	0.555500	0.004800	0.000500	0.000320
100	0.455500	0.003800	0.000500	0.000320
